# Effects of Different Heating Treatments on the Antioxidant Activity and Phenolic Compounds of Ecuadorian Red Dacca Banana

**DOI:** 10.3390/plants12152780

**Published:** 2023-07-27

**Authors:** Diego Armando Tuárez-García, Hugo Galván-Gámez, Cyntia Yadira Erazo Solórzano, Carlos Edison Zambrano, Raquel Rodríguez-Solana, Gema Pereira-Caro, Mónica Sánchez-Parra, José M. Moreno-Rojas, José L. Ordóñez-Díaz

**Affiliations:** 1 Faculty of Industry and Production Sciences, State Technical University of Quevedo, Av. Walter Andrade, km 1.5 Via Santo Domingo, Quevedo 120301, Ecuador; dtuarez@uteq.edu.ec (D.A.T.-G.); cerazo@uteq.edu.ec (C.Y.E.S.); 2 Department of Agrifood Industry and Food Quality, Andalusian Institute of Agricultural and Fisheries Research and Training (IFAPA), Alameda del Obispo, Avda Menéndez Pidal s/n, 14004 Córdoba, Spain; hugo.galvan@juntadeandalucia.es (H.G.-G.); raquel.rodriguez.solana@juntadeandalucia.es (R.R.-S.); mariag.pereira@juntadeandalucia.es (G.P.-C.); monica.sanchez.parra@juntadeandalucia.es (M.S.-P.); 3 Faculty of Business Sciences, State Technical University of Quevedo, Av. Walter Andrade, km 1.5 Via Santo Domingo, C.P. 73, Quevedo 120301, Ecuador; cezambrano@uteq.edu.ec; 4 MED—Mediterranean Institute for Agriculture, Environment and Development & CHANGE—Global Change and Sustainability Institute, Faculdade de Ciências e Tecnologia, Universidade do Algarve, Campus de Gambelas, 8005-139 Faro, Portugal; 5 Foods for Health Group, Instituto Maimónides de Investigación Biomédica de Córdoba (IMIBIC), 14004 Córdoba, Spain

**Keywords:** banana, *Musa*, boiling, roasting, baking, thermal process, polyphenols, flavonoids, Antioxidants, HPLC-HRMS

## Abstract

The banana is a tropical fruit characterized by its composition of healthy and nutritional compounds. This fruit is part of traditional Ecuadorian gastronomy, being consumed in a wide variety of ways. In this context, unripe Red Dacca banana samples and those submitted to different traditional Ecuadorian heating treatments (boiling, roasting, and baking) were evaluated to profile their phenolic content by ultra-high-performance liquid chromatography coupled to high-resolution mass spectrometry (UHPLC-HRMS) and the antioxidant activity by ORAC, ABTS, and DPPH assays. A total of sixty-eight phenolic compounds were identified or tentatively identified in raw banana and treated samples, highlighting the content in flavonoids (flavan-3-ols with 88.33% and flavonols with 3.24%) followed by the hydroxybenzoic acid family (5.44%) in raw banana samples. The total phenolic compound content significantly decreased for all the elaborations evaluated, specifically from 442.12 mg/100 g DW in fresh bananas to 338.60 mg/100 g DW in boiled (23.41%), 243.63 mg/100 g DW in roasted (44.90%), and 109.85 mg/100 g DW in baked samples (75.15%). Flavan-3-ols and flavonols were the phenolic groups most affected by the heating treatments, while flavanones and hydroxybenzoic acids showed higher stability against the heating treatments, especially the boiled and roasted samples. In general, the decrease in phenolic compounds corresponded with a decline in antioxidant activity, evaluated by different methods, especially in baked samples. The results obtained from PCA studies confirmed that the impact of heating on the composition of some phenolic compounds was different depending on the technique used. In general, the heating processes applied to the banana samples induced phytochemical modifications. Even so, they remain an important source of bioactive compounds for consumers.

## 1. Introduction

The banana (*Musa* sp.) is one of the most widely consumed fruits worldwide, with 125 million tons being produced around the globe [1]. It should be noted that, in some areas of South America, Africa, and Asia, this product is an essential part of their gastronomy. According to FAO data for 2021, Ecuador had 164,085 ha of harvest area, ranking tenth worldwide. Nevertheless, this country was the fifth world producer of bananas, and the second in South America, reaching 6,680,000 tons (5.3% of the world production). These data show that the production/harvest area ratio in Ecuador was better than in other countries such as the United Republic of Tanzania, Rwanda, Philippines, Peru, Angola, or the Democratic Republic of the Congo.

Ecuador has a great climatic diversity where different varieties of bananas have been able to adapt. In this context, a wide genetic diversity has developed due to different natural crosses between banana species and varieties, giving rise to new varieties with interesting nutritional and organoleptic properties for consumers [2]. Among them, the Red Dacca banana (*Musa* sp. AAA) is a variety that stands out because of its purple or reddish peels and for being sweeter than common bananas. From the agronomic point of view, this variety is cultivated in high areas due to its specific climate and soil requirements, resisting low temperatures and fungal diseases. Additionally, this variety has a shorter harvest period (7–8 weeks after inflorescence) [3]. All these aspects reduce production costs, making its cultivation highly attractive for small and medium producers.

The banana is a tropical fruit characterized by its nutritional composition, mainly for its content of dietary fiber, resistant starch, minerals (potassium, calcium, manganese, magnesium, iron), and vitamins (folate, niacin, riboflavin, and B6) [4]. In relation to its pectin content, banana consumption has been associated with maintaining blood glucose levels, antiulcerogenic and antidiarrheal activity, or alleviating intestinal pain [5]. Furthermore, the banana is a source of phenolic acids and flavonoids, such as flavan-3-ols, flavanones, and flavonols [6,7]. Generally, fruits rich in phenolic compounds have been related to potential health benefits for consumers. These molecules are antioxidant compounds with the ability to protect against oxidative damage caused by reactive oxygen species related to human diseases, such as degenerative diseases, cardiovascular diseases, chronic inflammation, or cancer [8,9].

Nowadays, the research in food science and nutrition is not only focused on the nutritional composition of simple or raw products, since it is necessary to evaluate the nutritional and health components of derived products, including cooked foods, processed foods, or food preparations [10,11]. Bananas are consumed in a wide variety of ways in Ecuador, including as fresh, roasted, or baked products. Especially unripe banana flour has been proposed as a functional ingredient for the elaboration of breads, cookies, or pasta [12]. This product is usually elaborated by cutting the fruit into slices and drying it in an oven for several hours [13]. In general, these cooking processes induce changes in their organoleptic characteristics, including modifications to the physical and chemical composition of banana products [14]. In this context, changes in bioactive compounds are very complex, depending on a wide diversity of factors such as ripening stage, cooking elaboration, and temperature, among others. Thus, culinary elaboration may cause the degradation of thermolabile compounds or the formation of new ones by heat-induced chemical reactions [15]. In this instance, phenolic compounds, as well as antioxidant activity, can be affected by thermal processes [15]. In the literature, we can find several studies on the effect of heating treatments on antioxidant activity and phenolic compounds in bananas, boiling being the most investigated cooking method. Passo-Tsamo et al. [14] evaluated the effect of the boiling process on phenolic compounds (hydroxycinnamic acids and flavanols) in six plantain banana varieties. Borges et al. [16] analyzed only five phenolic aglycones compounds in two cooked banana genotypes by boiling, microwaving, and stir-frying. Currently, the information reported on the effect of different heat treatments on specific phenolic compounds is limited and not clear-cut.

Therefore, due to the limited information found in the literature regarding exhaustive phenolic compound content and profile in traditional cooking elaborations of bananas, the aim of this work was to study the impact in the Red Dacca banana pulp (*Musa* sp. AAA) of three traditional Ecuadorian heating treatments (boiling, roasting, and baking) on the antioxidant activity and phenolic compound profiles using ultra-high-performance liquid chromatography coupled to high-resolution mass spectrometry (UHPLC-HRMS).

## 2. Results and Discussion

### 2.1. Phenolic Compounds Profile

A total of sixty-eight phenolic compounds were identified and quantified in raw and heat treatment banana samples, including nine flavan-3-ols, nine flavanones, thirteen flavonols, sixteen hydroxybenzoic acids, sixteen hydroxycinnamic acids, and five phenylacetic acids. Table S1 (Supplementary Information) shows the characteristics for the identification of compounds, such as retention time (RT), the experimental accurate mass, and the error (ppm) between the exact accurate mass and the mass found of the detected compounds. The family of flavan-3-ols showed the highest concentration of phenolic compounds in the raw banana samples, reaching 390.51 mg/100 g DW (88%). The composition of this group consisted mainly of dimers and trimers of procyanidin and (−)-epicatechin (Table 1). In general, most of the literature has focused the research of flavan-3-ols on monomers, such as (−)-epicatechin, (+)-catechin, or (−)-epigallocatechin [8,14,17,18], neglecting the contribution of flavan-3-ols polymers in the banana pulp. In this sense, some publications have previously described (−)-epicatechin as the main flavonoid in green banana flour [17], whilst procyanidins were determined in high concentrations in banana peels [19]. Concerning our data, the content and profile of the flavan-3-ols group found in the banana pulp are not in agreement with most of the literature since we note a higher contribution of flavan-3-ols polymers. Other flavonoids were identified in bananas samples, including flavanones, eriodyctiol hexosides being the most abundant, and flavonols, where quercetin dihexoside and myricetin rutinoside were the compounds with the highest concentrations. Regarding the hydroxybenzoic acids group, 4 hydroxy-3-methoxybenzoic acid and 3-hydroxy-4-methoxybenzoic acid compounds showed the highest contents, whilst cinnamic acid, 3-(4′-hydroxyphenyl)propanoic acid), and 4′-hydroxy-3′-methoxycinnamic acid hexoside were the main compounds of the hydroxycinnamic acids group. Concerning the phenylacetic acids family, 4′-hydroxy-3′-methoxyphenylacetic acid was the main compound, followed by 3,4-dihydroxyphenylacetic acid and 3′-hydroxy-4′-methoxyphenylacetic acid. Generally, the total content and profile of phenolic compounds described in the literature are very variable. This evidence could be attributed to different considerations such as pre-harvest (varieties, species, cropping areas, etc.) and post-harvest factors (ripening, preservation, and cooking, among others). Moreover, most of the studies in the literature were focused on estimating the total phenolic content of banana samples using spectrophotometry techniques, estimating the total content with respect to gallic acid (3,4,5-Trihydroxybenzoic acid) [7,20].

Regarding the impact of the heating treatments used in our study, the total content of phenolic compounds significantly decreased for all the elaborations evaluated, from 442.12 mg/100 g DW in raw banana to 338.60, 243.63, and 109.85 mg/100 g DW in the boiled (23.41%), roasted (44.90%), and baked (75.15%) samples, respectively (Table 1). The literature reports a high variability of the effects of cooking on the content of phenolic compounds in fruits and vegetables, sometimes being positive [15,21] and others not [6,21]. These results show that the different heating treatments tested significantly affect the content of phenolic compounds in bananas. Additionally, the decrease in phenolic compounds in boiled samples can be explained by an extraction process that takes place during boiling. In the literature, it is demonstrated that the previously mentioned impact increases when the banana is boiled without the peel [16].

**Figure 1:**
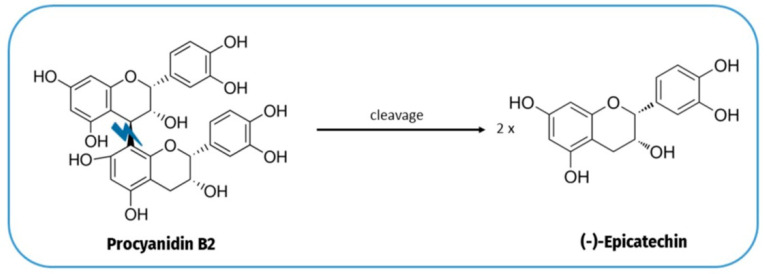
Chain cleavage of procyanidins in flavan-3-ol monomers.

The content of flavan-3-ols significantly decreased for all preparations, being lower in the boiled samples (286.63 mg/100 g DW) and more pronounced in the baked ones (74.45 mg/100 g DW). This decrease was mainly related to the degradation of dimers and trimers of procyanidin (Table 1). Additional decreases were observed for (+)-catechin, (−)-epigallocatechin, or (+)-gallocatechin. Borges et al. [14] observed that (+)-catechin decreased in *Pelipita* and *D’Angola* banana varieties after boiling. It was observed that (−)-epicatechin significantly increased in boiled samples, while significantly decreased for the other treatments (roasted and baked bananas). Hence, these results could suggest that procyanidins could be degraded into (−)-epicatechin to some extent (Figure 1). The formation of flavan-3-ols monomers from procyanidins has been confirmed previously in cooking research [22]. Therefore, the cooking temperature can be directly related to the chain cleavage of procyanidins [23].

**Figure 2:**
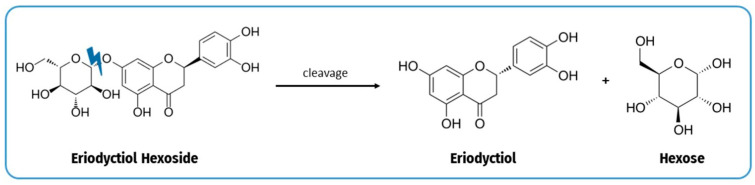
Cleavage of the monosaccharide from eriodyctiol hexoside producing the aglycone during the heat treatment.

Total flavanones represented 0.31% of the total phenolics of fresh bananas. No significant changes were observed in the concentration of this family of compounds between the raw samples and the samples submitted to heat treatments, while a slight impact was observed among the boiling and roasting treatments and baking ones (Table 1). In addition, changes in individual flavanone content were observed in different elaborations, mainly due to changes in the concentration of naringenin and eriodyctiol hexosides. Hexoside isomer II increased significantly in the boiled and roasted samples, remaining stable in the baked samples. On the other hand, it was observed that eriodyctiol aglycone increased in the roasted and baked samples, probably due to the fragmentation of the monosaccharide from eriodyctiol hexoside isomers during the heating treatment (Figure 2). Although a similar thermohydrolysis phenomenon seems to occur for the naringenin hexosides, such as naringenin-7-glucoside or naringenin hexoside I and III, naringenin aglycone were not detected after the heating processes. This could be due to additional cleavage reactions of the core compounds. The thermohydrolysis process is a chemical reaction widely described in the literature [6,15,19].

The total concentration of flavonols represented 3.24% of the pulp of the raw banana, and it was affected by all the heat treatments (Table 1), passing from 14.33 mg/100 g DW in the raw banana to 12.47 mg/100 g DW in the boiled samples (13%), to 6.67 mg/100 g DW in the roasted samples (53%) and, finally, to 2.90 mg/100 g DW in the baked samples (80%). Although most of the flavonols significantly decreased during the transformation treatments, this was most noticeable for quercetin dihexoside and myricetin rutinoside. Similar observations were highlighted by some authors regarding quercetin derivatives (rutin and quercetin deoxyhexose-hexoside) in boiled banana peels [14]. The myricetin rutinose content decreased from 6.32 mg/100 g DW to 5.54, 2.60, and 0.99 mg/100 g DW in the boiled, roasted, and baked samples, respectively. In contrast, Passo-Tsamo et al. [14] obtained different results for myricetin deoxyhexose-hexoside, where no significant differences were observed in boiled banana pulps and peels. Other flavonol hexosides, such as quercetin hexosides or kaempferol 3-rutinoside, showed a similar trend, decreasing their content after heat treatments (Table 1).

The total content of the hydroxybenzoic acids group represented 5.44% of the raw banana pulp and this group of compounds showed higher stability against the heating treatments tested. In our study, statistically significant differences only being observed between the roasted and baked samples (24.97 vs. 20.78 mg/100 g DW, respectively) (Table 1). In this context, 4-hydroxy-3-methoxybenzoic and 3-hydroxy-4-methoxybenzoic acids were the main compounds of the group, representing 70 and 24.2% of the raw banana pulp, respectively. 4-hydroxy-3-methoxybenzoic acid maintained a stable concentration during all the heat treatments. On the contrary, the 3-hydroxy-4-methoxybenzoic acid content was heavily affected by the treatments, with an important decrease for the baked samples and, to a lesser degree, in the roasted samples. On the other hand, it was observed that 3,4,5-Trihydroxybenzoic acid, 3,4,5-Trihydroxybenzoic acid hexoside, and 4-hydroxybenzoic acid increased during the preparations, especially in the roasted and baked samples. The increase of 3,4,5-Trihydroxybenzoic acid and hydroxybenzoic acids after cooking elaborations has been observed in previous studies [16].

Differences in the effects of heating treatments were observed in the total content of hydroxycinnamic acids (representing 1.69% of the fresh banana pulp). The concentration of this group increased in the boiled (9.19 mg/100 g DW) and roasted (9.09 mg/100 g DW) samples, while the baked (7.40 mg/100 g DW) samples remained unchanged (Table 1). Considering the individual profile, the compounds of this group had a different evolution depending on the treatment. The boiled samples showed a significant increase in 3′,4′-dihydroxycinnamic acid, caffeoyl hexoside isomer I, and 4′-hydroxy-3′-methoxycinnamic acid hexoside. Similarly, cinnamic acid, 4′-hydroxy-3′-methoxycinnamic acid, 3′-hydroxy-4′-methoxycinnamic acid, and 4′-hydroxy-3′-methoxycinnamic acid hexoside increased their concentrations in the roasted samples. Differences in profile were also observed between the fresh and baked bananas, although the total content did not vary. In this case, cinnamic and 3′-hydroxy-4′-methoxycinnamic acid compounds increased in the baked samples, whilst caffeoyl hexoside isomers, 4′-hydroxy-3′-methoxycinnamic acid conjugate, and syringin were higher in the raw banana. In the literature, some authors have reported changes in the content of hydroxycinnamic acids in banana pulp after cooking, mainly 4′-Hydroxy-3′-methoxycinnamic acid hexoside in *Moto Ebanga* cultivar [14]. They observed an increase in 4′-Hydroxy-3′-methoxycinnamic acid from the cleavage of their hexoside, similar to that observed in roasted samples (Table 1). Additionally, the significant increase in some hydroxycinnamic acids, such as cinnamic acids in roasted and boiled samples or 3′,4′-dihydroxycinnamic acid in boiled samples, may be due to the release of bound phenolics [24].

The total content of phenylacetic acid derivates was stable for the boiled and roasted samples, observing a significant drop in the baked ones. Specifically, the content of 3-hydroxyphenylacetic acid decreased from 0.38 to 0.14 mg/100 g DW and 4′-hydroxy-3′-methoxyphenylacetic acid from 1.62 to 1.10 mg/100 g DW, namely 62% and 32%, respectively. Other compounds also declined, such as phenylacetic acid from 0.65 to 0.51 mg/100 g DW (22%), 3,4-dihydroxyphenylacetic acid from 1.02 to 0.81 mg/100 g DW (21%), and 3′-hydroxy-4′-methoxyphenylacetic acid from 0.75 to 0.65 mg/100 g DW (13%).

### 2.2. Multivariate Analyses: Principal Component Analysis (PCA)

**Figure 3:**
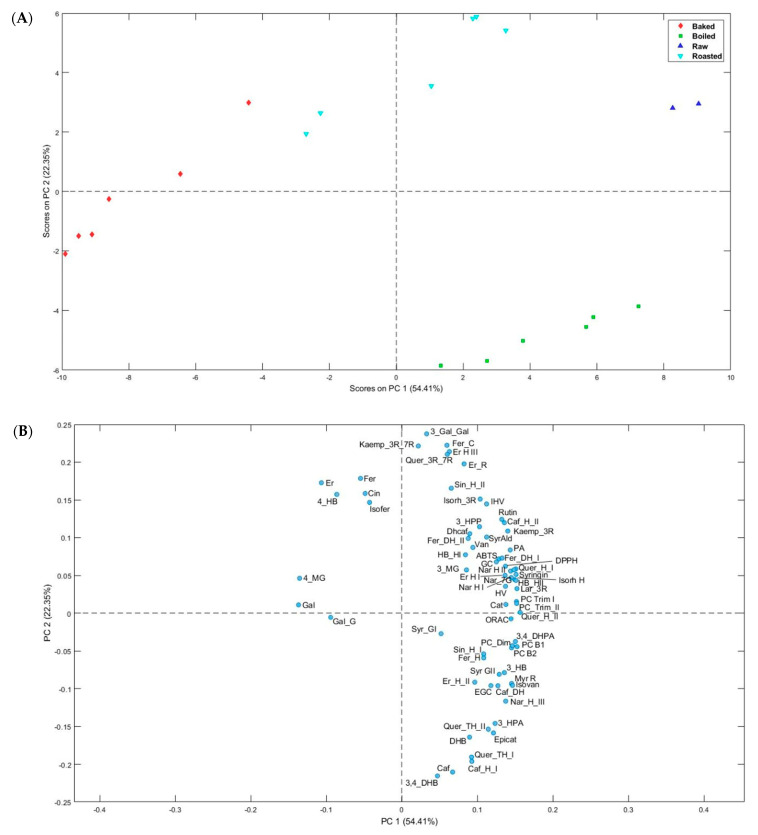
Principal component analysis (PCA) scores (**A**) and loadings (**B**) plots using phenolic compounds content of banana heating treatments. Cat: (+)-Catechin; Epicat: (−)-Epicatechin; EGC: (−)-Epigallocatechin; GC: (+)-Gallocatechin; PC_B2: Procyanidin_B2; PC_B1: Procyanidin_B1; PC_Dim: Procyanidin_dimer; PC_Trim_I: Procyanidin_trimer_I; PC_Trim_II: Procyanidin_trimer_II; Er: Eriodyctiol; Er_R: Eriodyctiol-rutinoside; Er_H_I: Eriodyctiol_hexoside_I; Er_H_II: Eriodyctiol_hexoside_II; Er_H_III: Eriodyctiol_hexoside_III; Nar_H_I: Naringenin-Hexoside_I; Nar_H_II: Naringenin-Hexoside_II; Nar_7G: Naringenin-7-glucoside; Nar_H_III: Naringenin-Hexoside_III; Quer_H_I: Quercetin-hexoside_I; Quer_DH: Quercetin-Dihexoside; Quer_H_II: Quercetin-hexoside_II; Quer_TH_I: Quercetin-Trihexoside_I; Quer_TH_II: Quercetin-Trihexoside_II; Quer_3R_7R: Quercetin_3-Rutinoside_7-Rhamnoside; Rutin: Rutin; Kaemp_3R_7R: Kaempferol_3-Rutinoside-7-Rhamnoside; Kaemp_3R: Kaempferol-3-rutinoside; Isorh_3R: Isorhamnetin-3-rutinoside; Isorh_H: Isorhamnetin-hexoside; Myr_R: Myricetin_rutinoside; Lar_3R: Laricitrin_3-rutinoside; Gal: 3,4,5-Trihydroxybenzoic acid; 3_MG: 3,4-dihydroxy-5-methoxybenzoic acid; 4_MG: 3,5-dihydroxy-4-methoxybenzoic acid; HB_HI: Hydroxybenzoic_acid_hexoside_I; HB_HII: Hydroxybenzoic_acid_hexoside_II; Syr_GI: 3,5-dimethoxy-4-hydroxybenzoic acid hexoside_I; Syr_GII: 3,5-dimethoxy-4-hydroxybenzoic acid hexoside_II; Gal_G: Galloyl-glucose; 4_HB: 4-Hydroxybenzoic_acid; 3_HB: 3-Hydroxybenzoic_acid; 3,4_DHB: 3,4-Dihydroxybenzoic_acid; DHB: Dihydroxybenzoic_acid; 3_Gal_Gal: 3-Galloyl-Gallic_Acid; SyrAld: Syringaldehyde; Van: 4-Hydroxy-3-methoxybenzoic acid; Isovan: 3-Hydroxy-4-methoxybenzoic acid; Fer_H: 4′-Hydroxy-3′-methoxycinnamic acid hexoside; Fer_DH_I: 4′-Hydroxy-3′-methoxycinnamic acid dihexoside_I; Fer_DH_II: 4′-Hydroxy-3′-methoxycinnamic acid dihexoside_II; Fer_C: 4′-Hydroxy-3′-methoxycinnamic acid conjugate; Caf: 3′,4′-dihydroxycinnamic acid; Caf_DH: Caffeoyl-Dihexoside; Caf_H_I: Caffeoyl-hexoside_I; Caf_H_II: Caffeoyl-hexoside_II; Sin_H_I: Sinapic_acid_hexoside_I; Sin_H_II: Sinapic_acid_hexoside_II; Fer: 4′-Hydroxy-3′-methoxycinnamic acid; Isofer: 3′-hydroxy-4′-methoxycinnamic acid; Cin: Cinnamic_acid; Dhcaf: 3-(3′,4′-dihydroxyphenyl)propanoic acid; 3_HPP: 3-(4′-Hydroxyphenyl)propanoic_acid; Syringin: Syringin; PA: Phenylacetic_acid; 3_HPA: 3-Hydroxyphenylacetic_acid; HV: 4′-hydroxy-3′-methoxyphenylacetic acid; IHV: 3′-hydroxy-4′-methoxyphenylacetic acid; 3,4_DHPA: 3,4-Dihydroxyphenylacetic acid.

A multivariate analysis, principal component analysis (PCA), was carried out to evaluate the effect of the heating treatments on the concentration and profile of phenolic compounds and antioxidant activities in raw bananas and three elaborations. We sampled the raw banana before the heating treatments, and these samples were analyzed by duplicate. Every heating treatment was performed by triplicate and each sample obtained was analyzed by duplicate (for a total of six data per treatment). Figure 3 shows the corresponding scores (A) and loadings (B) plotted using the first two principal components, explaining 76.76% of the cumulative variance. PC1 (54.41% of the data variability) shows that the samples were separated according to a higher content of phenolic compounds. Thus, the raw banana samples were placed on the right side of the scores plot, highly related to most of the phenolic compounds found, followed by boiled and roasted banana samples (Figure 3B). Most of the flavan-3-ols (mainly dimers and trimers) and flavonols were highly related to the raw samples. These flavonoid families were sensible to heat treatments, decreasing in the three types of cooking. Boiling and roasting had a lower impact on the phenolic content than baking. The antioxidant activity methods (ABTS, DPPH, and ORAC) were placed on the right side, and highly correlated with most phenolic compounds. Additionally, the compounds more related to baking (middle-left part) were 3,4,5-Trihydroxybenzoic acid, 3,5-dihydroxy-4-methoxybenzoic acid, 3,4,5-Trihydroxybenzoic acid hexoside, and 4 hydroxybenzoic acid, belonging to the family of hydroxybenzoic acids, as well as eriodyctiol, which increased significantly after the heating treatment. On the other hand, PC2 tends to separate the boiled samples (bottom right part) from the rest (Figure 3A). The boiled samples were highly related to dihydroxybenzoic acid isomers, 3′,4′-dihydroxycinnamic acid, caffeoyl hexoside isomer I, quercetin trihexoside isomers, and (−)-epicatechin. These compounds significantly increased in the banana samples after boiling. The fresh and roasted samples are in the positive area of PC2 (upper middle-right part), correlated with 3-galloyl-gallic acid, 4′-hydroxy-3′-methoxycinnamic acid conjugate, eriodyctiol hexoside isomer III, quercetin 3-rutinoside-7-rhamnoside, kaempferol 3-rutinoside-7-rhamnoside, and eriodyctiol rutinoside. These compounds were quantified in the raw samples and most remained stable after roasting. Regarding antioxidant activity, ORAC was tending toward the center line in PC2, since it does not present significant differences between raw, baked, and roasted samples. However, ABTS and DPPH were placed in the upper middle-right part and were correlated with raw and roasted samples. In general, these results indicate that the impact on the composition of some phenolic compounds differed for each heating treatment, allowing the different groups on the PCA to be separated.

### 2.3. Antioxidant Activity

**Figure 4:**
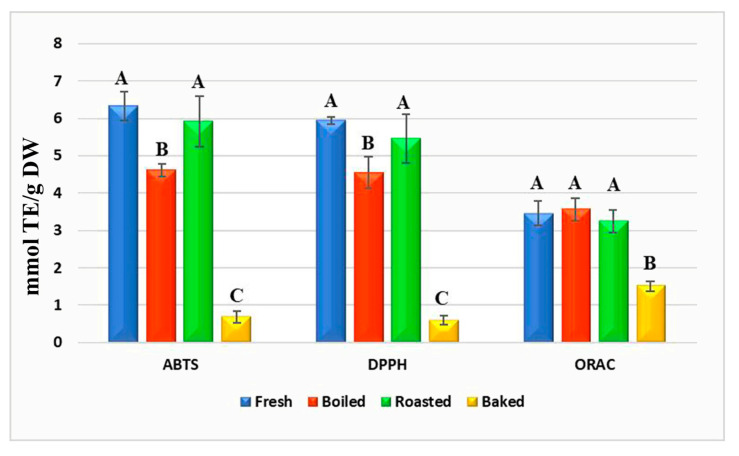
Antioxidant activity evaluated by ABTS, DPPH, and ORAC assays of fresh, boiled, roasted, and baked banana samples. Different uppercase letters on the top of each bar showed significant differences (*p* < 0.05) within the different products as measured by a one-way ANOVA followed by the Tukey post hoc test.

The antioxidant activity of the raw, boiled, roasted, and baked banana samples was evaluated using several assays, such as ABTS, DPPH, and ORAC (Figure 4). Regarding the ABTS test, the values from samples of fresh and roasted samples did not show differences, whilst the boiled banana samples were significantly lower. In particular, the antioxidant activity of the baked samples drastically decreased in ABTS assays. Similar results were observed in the DPPH test, where the antioxidant activity of the roasted samples remained stable, while that of the boiled and baked samples declined. Results from the ORAC method were slightly different since the antioxidant activity of the baked samples significantly decreased, but no differences were observed between the fresh, boiled, and roasted samples. These differences in the results of antioxidant activity methods are due to different chemical principles, namely ABTS and DPPH assays which are based on the antioxidant activity of sample extracts to scavenge the free-radical cation, while the ORAC method evaluates peroxyl-radical scavenging [17,25,26,27]. In general, all the methods found that the antioxidant activity of the baked samples was highly affected. These results could be explained due to this product presenting a significant drop in phenolic compounds (from 442.12 to 109.85 mg/100 g DW), which play an important role in the antioxidant activity of these products. In general, thermal treatment can cause the degradation of phenolic compounds and other bioactive compounds, such as organic acids, vitamins, etc., affecting antioxidant activity [15,28].

## 3. Materials and Methods

### 3.1. Chemicals

Formic acid (FA), phosphoric acid (85%), acetone, HPLC–MS (Liquid Chromatography Mass Spectrometry) grade acetonitrile, HPLC–MS grade water, and HPLC–MS grade methanol were acquired from Panreac (Barcelona, Spain). Potassium dihydrogen phosphate, sodium hydrogen carbonate, and potassium hydrogen phosphate were purchased from VWR International Eurolab (Barcelona, Spain). Reference phenolic standard compounds, including 3,4,5-Trihydroxybenzoic acid, 3,4-dihydroxy-5-methoxybenzoic acid, 3,5-dihydroxy-4-methoxybenzoic acid, 4-hydroxybenzoic acid, 3-hydroxybenzoic acid, 4-hydroxy-3 methoxybenzoic acid, 3-hydroxy-4-methoxybenzoic acid, 3′,4′-dihydroxycinnamic acid, 4′-hydroxy-3′-methoxycinnamic acid, cinnamic acid, 3-(3′,4′-dihydroxyphenyl)propanoic acid, 3-(4′-hydroxyphenyl)propanoic acid, phenylacetic acid, 3 hydroxyphenylacetic acid, 4′-hydroxy-3′-methoxyphenylacetic acid, 3′ hydroxy-4′-methoxyphenylacetic acid, 3,4-dihydroxyphenylacetic acid, 3,4-dihydroxybenzoic acid, (−)-epigallocatechin, procyanidin B2, (+)-catechin, (−)-epicatechin, rutin, kaempferol-3-rutinoside, naringenin-7-glucose, myricetin, and naringenin, were purchased from Sigma-Aldrich (Steinheim, Germany). 2,2′-Diazo-bis-amidinepropane-dihydrochloride (AAPH), 6-hydroxy-2,5,7,8-tetramethylchroman-2-carboxylic acid (trolox), fluorescein, potassium hydroxide, sodium hydroxide, potassium persulfate, 2,2-diphenyl-1-picrylhydrazyl (DPPH), and 2,2′-azino-bis(3-ethylbenzothiazoline-6 sulfonic acid) diammonium salt (ABTS) were obtained from Sigma-Aldrich (Steinheim, Germany).

### 3.2. Materials and Samples Preparation

Red Dacca banana (*Musa* sp. AAA) samples were provided from a field of bananas in “el Cantón la Maná” (Cotopaxi, Ecuador) planted in 2019. Banana bunches were harvested in February 2022, and the harvesting criterion was selecting fruits with a diameter of 39–46 mm around 8 weeks after the inflorescence. The samples complied with all the postharvest quality controls, and they were packed in cardboard boxes of approximately 6.9 kg. All samples were stored at room temperature (25 ± 2 °C) and 70 ± 5% relative humidity until processing. The samples were stored for 15 days from the harvest until the elaborations. Additionally, the maturity of the bananas (unripe bananas) was the same for the raw samples and subsequent heat treatments.

**Figure 5:**
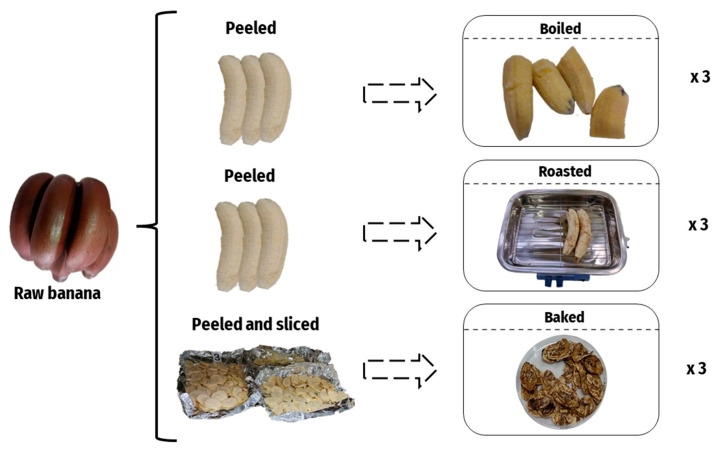
Graphical scheme of the sampling design.

In order to evaluate the impact of the heating treatments on the bioactive compounds, the banana samples were cooked with different preparation methods, including boiling, roasting, and baking (Figure 5). All the analyses were conducted on peeled banana samples.

Boiled preparations were performed by boiling the bananas in 500 mL of drinking water for 15 min in a covered stainless-steel pot heated on a laboratory hotplate RH digital KT/C (IKA^®^, Staufen, Germany). Previously, the temperature was controlled to reach 100 °C before introducing the samples into the pot. For the roasted elaboration, a covered grill was used to cook the banana samples for 20 min at 120 °C. The samples were turned for a homogeneous elaboration. For the baking process, the banana samples were sliced and dried in an oven (Selecta Digitheat 2001243, Barcelona, Spain) for 2 h at 100 °C, turning the samples for a homogeneous elaboration.

All the elaborations were performed by triplicate, and each repetition was formed by two peeled bananas. The final samples were cut into small pieces, lyophilized in a freeze dryer ECO EVO (Tred Technology S.R.L., Ripalimosani, Italy), ground, and stored at −80 °C until analysis.

### 3.3. Extraction of Phenolic Compounds

The extraction of polyphenols from banana samples was adapted from Ordóñez-Díaz et al. [27] with some modifications. Briefly, 200 mg of the sample was mixed with 1 mL of a methanol/acidified water mixture (80:20, *v*/*v*) with 1% formic acid. The samples were sonicated using an ultrasonic bath (Selecta, Barcelona, Spain) for 10 min and centrifuged at 15,000 rpm for 15 min at 4 °C using a microcentrifuge (Eppendorf, Hamburg, Germany), and finally, supernatants were collected. The pellet was re-extracted with 1 mL of the same solvent as described above. All the supernatants were pooled and made up to a final volume of 2 mL and stored at −80 °C until analysis.

### 3.4. Antioxidant Activity Analysis

The antioxidant activity of the banana samples was analyzed using three different assays: ABTS (2,2′-azino-bis(3-ethylbenzothiazoline-6-sulfonic acid) diammonium salt), DPPH (2,2-diphenyl-1-picrylhydrazyl), and ORAC (Oxygen Radical Absorbance Capacity) methods. The antioxidant activity analyses were performed using a Synergy HTX Multi-Mode Microplate Reader (Biotek Instruments, Winooski, VT, USA).

#### 3.4.1. ABTS Method

Free radical ABTS scavenging activity was assessed in the phenolic extract following the methods previously described by Ordóñez-Díaz et al. [29]. The antioxidant activity results were expressed as mmol of Trolox equivalents per 100 g of dry weight (mmol TE/100 g DW). The ABTS analyses were performed in triplicate.

#### 3.4.2. DPPH Method

The free radical scavenging activity was measured using the DPPH method adapted for the microplate reader [29]. The antioxidant activity results were expressed as mmol of Trolox equivalents per 100 g of dry weight (mmol TE/100 g DW). The DPPH analyses were performed in triplicate.

#### 3.4.3. ORAC Method

The oxygen radical scavenging activity in the phenolic extractions was analyzed by the ORAC assay according to the method previously published by Madrona et al. [30] and modified by Pereira-Caro et al. [31]. Briefly, 25 µL of banana samples or standard solutions (Trolox) are mixed with 150 µL of fluorescein solution (8.5 × 10^−5^ mM) prepared in 75 mM of phosphate buffer (pH 7.4) and 30 µL of AAPH (153 mM) as a peroxyl radical generator. The fluorescence was measured every 90 s for 90 min at 485 and 528 nm excitation and emission wavelengths, respectively. The ORAC values were expressed as mmol Trolox equivalents per gram of dry sample (mmol TE/g DW). The ORAC analyses were performed in triplicate.

### 3.5. Phenolic Compound Analysis

The identification and quantification of the phenolic compounds in the fresh bananas and their products were performed by using a UHPLC-HRMS mass spectrometer system (Thermo Scientific, San José, CA, USA) comprised of a UHPLC pump and an autosampler operating at 10 °C (ThermoFisher Scientific, San Jose, CA, USA). The column was a Zorbax SB-C18 RRHD column (100 × 2.1 mm i.d., 1.8 µm (Agilent, Santa Clara, CA, USA)), and a guard pre-column of the same stationary phase was used. The injection volume was 5 μL and the separation was obtained at a flow rate of 0.2 mL/min at 40 °C with the gradient program employed by Ordóñez-Díaz et al. [27].

The column eluate went directly to an Exactive Orbitrap mass spectrometer (Thermo Scientific, San José, CA, USA) fitted with a heated electrospray ionization probe (HESI) operating in negative ionization mode for the determination of phenolic compounds. Full scans were recorded in *m*/*z* range from 100 to 1200 with a resolution of 50,000 Hz and with a full AGC target of 100,000 charges, using 2 microscans. Analyses were also based on scans with in-source collision-induced dissociation (CID) at 25.0 eV. The capillary temperature of the MS experiment with HESI in negative ionization mode was 320 °C, the sheath gas was 40 units, the heater temperature was 375 °C, the auxiliary gas was 10 units, and the spray voltage was 3.0 kV. Data acquisition and processing were carried out using Xcalibur 3.0 software (Thermo Scientific, San José, CA, USA). Phenolic compounds were identified comparing the exact mass and the retention time with available standards. When standards were not available, phenolic compounds were tentatively identified by comparing the theoretical exact mass of the molecular ion and the experimental accurate mass of the molecular ion, as well as by searching in several chemical databases, such as Metlin, Phenol Explorer, PubChem, or Phytohub. Phenolic compounds having molecular masses within the prespecified tolerance (≤5 ppm) of the query masses were retrieved from these databases. The quantification was carried out using standard references of 0.01–100.0 mg/L calibration curves. In the absence of reference standards, these compounds were quantified using a calibration curve of a closely related parent compound. The limits of detection (LOD) and quantification (LOQ) varied from 0.003 to 0.48 mg/L and from 0.01 to 1.6 mg/L, respectively.

### 3.6. Statistical Analysis

Univariate statistical analyses were performed to identify the differences among samples using Statistix v. 9.0 software. The data were subjected to an analysis of variance (ANOVA), followed by a comparison of means according to Tukey post hoc tests. The level of significance was established at *p* < 0.05.

A principal component analysis (PCA) was carried out as an unsupervised method to evaluate the effect of heat treatments on the profiles of phenolic compounds and antioxidant activity in different banana products. The PCA model was calculated with the PLS Toolbox 8.7 (Eigenvector Research Inc., Wenatchee, WA, USA) working under a MATLAB environment. This section may be divided into subheadings. It should provide a concise and precise description of the experimental results, their interpretation, as well as the experimental conclusions that can be drawn.

## 4. Conclusions

An evaluation of the impact on phenolic compounds and the antioxidant activity of bananas from the traditional cuisine of Ecuador subjected to three heating treatments, namely boiling, roasting, and baking (to obtain flour for further elaborations), was conducted. The heating processes applied to the banana samples induced phytochemical modifications, resulting in a decrease in the total content of phenolic compounds, from 442.12 mg/100 g DW in raw banana to 338.60, 243.63, and 109.85 mg/100 g DW in boiled, roasted, and baked samples, respectively. These changes were influenced by the sensitivity of each compound to be degraded under the heating process. Flavan-3-ols, flavonols, and hydroxycinnamic acids were the groups of phenolics most affected by the heating preparations, especially in the baked samples. Nevertheless, some compounds increased after treatments, such as (−)-epicatechin, eriodyctiol hexoside II, 3′,4′dihydroxycinnamic acid, caffeoyl hexoside I, and 4′-hydroxy-3′-methoxycinnamic acid hexoside in the boiled samples. The roasted banana samples showed a rise in eriodyctiol, eriodyctiol hexoside II, 4′-hydroxy-3′-methoxycinnamic acid, 3′-hydroxy-4′-methoxycinnamic acid, 3,4,5-Trihydroxybenzoic acid hexoside, 4 hydroxybenzoic acid, and cinnamic acid, whilst eriodyctiol, 3,4,5-Trihydroxybenzoic acid, 3,4,5-Trihydroxybenzoic acid hexoside, and 4 hydroxybenzoic acid increased in the baked samples. The antioxidant activity in different samples was highly related to the decrease in the content of phenolic compounds, especially in the baked samples where the antioxidant activity drastically decreased. In general, the different heating treatments had an important impact on the phenolic composition, the percentage decreases for the boiled and roasted samples being 23.41% and 44.90%, respectively, while a more pronounced effect was observed for the baked samples, with a 75.15% decrease. However, these traditional banana elaborations from unripe bananas remain an important source of bioactive compounds for consumers.

## Figures and Tables

**Table 1:** Phenolic compounds quantified in raw, boiled, roasted, and baked banana samples (mg/100 g DW).

Samples	Raw		Boiled		Roasted		Baked		*p*-Value
Flavan-3-ols									
(+)-Catechin	0.29	A	0.12	B	0.09	C	<LOD	D	***
(−)-Epicatechin	14.16	B	19.10	A	6.23	C	3.70	C	***
(−)-Epigallocatechin	0.146	AB	0.147	A	0.118	BC	0.114	C	***
(+)-Gallocatechin	0.133	A	0.123	AB	0.122	AB	0.115	B	*
Procyanidin B1	131.53	A	101.24	B	68.00	C	23.96	D	***
Procyanidin B2	104.64	A	68.21	B	38.81	C	15.02	D	***
Procyanidin dimer	20.61	A	14.24	B	9.49	C	5.20	D	***
Procyanidin trimer I	28.06	A	22.07	B	20.52	B	8.33	C	***
Procyanidin trimer II	90.94	A	61.38	B	53.98	C	18.01	D	***
Total	390.51	A	286.63	B	197.36	C	74.45	D	***
Flavanones									
Eriodyctiol	0.11	B	<LOD	C	0.29	A	0.32	A	***
Eriodyctiol hexoside I	0.17	A	0.09	B	0.09	B	0.03	C	***
Eriodyctiol hexoside II	0.56	C	1.20	A	0.92	B	0.60	C	***
Eriodyctiol hexoside III	0.033	A	0.011	B	0.026	A	0.014	B	***
Eriodyctiol rutinoside	0.23	A	0.08	C	0.16	B	0.08	C	***
Naringenin-7-glucoside	0.025	A	0.011	B	0.010	B	<LOD	C	***
Naringenin hexoside I	0.071	A	0.056	A	0.054	A	0.017	B	***
Naringenin hexoside II	0.12	A	0.10	B	0.10	AB	0.04	C	***
Naringenin hexoside III	0.033	A	0.031	A	0.011	B	0.007	C	***
Total	1.36	AB	1.57	A	1.65	A	1.10	B	***
Flavonols									
Quercetin hexoside I	0.026	A	0.016	B	0.015	B	0.007	C	***
Quercetin hexoside II	0.11	A	0.09	B	0.08	C	0.03	D	***
Rutin	0.29	A	0.21	B	0.25	A	0.15	C	***
Quercetin dihexoside	6.37	A	5.65	B	2.65	C	1.00	D	***
Quercetin 3-rutinoside 7-rhamnoside	0.05	AB	0.04	BC	0.06	A	0.04	C	***
Quercetin trihexoside I	0.005	B	0.025	A	0.005	B	0.002	C	***
Quercetin trihexoside II	0.010	B	0.020	A	0.009	B	0.005	C	***
Isorhamnetin hexoside	0.010	A	0.006	B	0.006	B	0.003	C	***
Isorhamnetin 3-rutinoside	0.009	A	0.007	B	0.008	A	0.006	B	***
Kaempferol 3-rutinoside	0.80	A	0.60	B	0.66	B	0.44	C	***
Kaempferol 3-rutinoside 7-rhamnoside	0.19	AB	0.13	B	0.21	A	0.16	B	***
Myricetin rutinoside	6.32	A	5.54	B	2.60	C	0.99	D	***
Laricitrin 3-rutinoside	0.14	A	0.13	A	0.13	A	0.07	B	***
Total	14.33	A	12.47	B	6.67	C	2.90	D	***
Hydroxybenzoic acids									
3,4,5-Trihydroxybenzoic acid	0.09	C	0.10	BC	0.11	B	0.18	A	***
3,4-dihydroxy-5-methoxybenzoic acid	0.049		0.050		0.050		0.048		ns
3,5-dihydroxy-4-methoxybenzoic acid	0.003	C	0.004	C	0.006	B	0.010	A	***
3,4,5-Trihydroxybenzoic acid hexoside	<LOD	C	0.048	B	0.056	A	0.059	A	***
3-Galloyl-gallic acid	0.17	A	0.09	C	0.18	A	0.11	B	***
Syringaldehyde	0.058		0.055		0.055		0.052		ns
4-Hydroxybenzoic acid	0.37	B	0.56	B	1.92	A	1.78	A	***
3-Hydroxybenzoic acid	0.12	A	0.12	A	0.11	B	0.10	B	***
Hydroxybenzoic acid hexoside I	0.20		0.19		0.19		0.18		ns
Hydroxybenzoic acid hexoside II	0.26	A	0.24	A	0.24	A	0.21	B	***
3,4-Dihydroxybenzoic acid	0.015	B	0.023	A	0.015	B	0.015	B	***
Dihydroxybenzoic acid	0.009	C	0.017	A	0.009	C	0.011	B	***
4-Hydroxy-3-methoxybenzoic acid	16.85		16.62		17.43		14.70		ns
3-Hydroxy-4-methoxybenzoic acid	5.83	A	6.36	A	4.58	B	3.30	C	***
3,5-dimethoxy-4-hydroxybenzoic acid hexoside I	0.007	C	0.016	A	0.015	A	0.011	B	***
3,5-dimethoxy-4-hydroxybenzoic acid hexoside II	0.015	AB	0.016	A	0.013	BC	0.011	C	***
Total	24.04	AB	24.51	AB	24.97	A	20.78	B	*
Hydroxycinnamic acids									
Cinnamic acid	2.67	C	3.19	C	4.09	A	3.64	B	***
3′,4′-dihydroxycinnamic acid	0.13	B	0.29	A	0.13	B	0.15	B	***
Caffeoyl hexoside I	0.37	B	1.11	A	0.21	C	0.19	C	***
Caffeoyl hexoside II	0.28	A	0.23	BC	0.24	B	0.21	C	***
Caffeoyl dihexoside	0.17	AB	0.19	A	0.16	B	0.15	B	***
4′-Hydroxy-3′-methoxycinnamic acid	0.14	B	0.15	B	0.20	A	0.18	AB	***
3′-hydroxy-4′-methoxycinnamic acid	0.04	C	0.05	BC	0.06	A	0.05	B	***
4′-Hydroxy-3′-methoxycinnamic acid hexoside	1.04	C	1.87	A	1.59	B	0.92	C	***
4′-Hydroxy-3′-methoxycinnamic acid dihexoside I	0.18		0.17		0.17		0.16		ns
4′-Hydroxy-3′-methoxycinnamic acid dihexoside II	0.16		0.16		0.16		0.15		ns
4′-Hydroxy-3′-methoxycinnamic acid conjugate	0.42	A	0.21	B	0.40	A	0.23	B	***
3-(4′-hydroxyphenyl)propanoic acid	1.21		0.94		1.05		0.81		ns
3-(3′,4′-dihydroxyphenyl)propanoic acid	0.03		0.03		0.03		0.03		ns
Syringin	0.30	A	0.27	A	0.26	A	0.23	B	***
Sinapic acid hexoside I	0.16	AB	0.18	A	0.16	AB	0.15	B	**
Sinapic acid hexoside II	0.17		0.16		0.17		0.16		ns
Total	7.46	B	9.19	A	9.09	A	7.40	B	***
Phenylacetic acids									
Phenylacetic acid	0.65	A	0.58	A	0.58	A	0.51	B	***
3-Hydroxyphenylacetic acid	0.38	A	0.41	A	0.17	B	0.14	B	***
3,4-Dihydroxyphenylacetic acid	1.02	A	1.00	A	0.90	B	0.81	C	***
4′-hydroxy-3′-methoxyphenylacetic acid	1.62	A	1.57	A	1.53	A	1.10	B	***
3′-hydroxy-4′-methoxyphenylacetic acid	0.75	A	0.68	AB	0.71	AB	0.65	B	*
Total	4.42	A	4.23	A	3.89	A	3.22	B	***
Total phenolic compounds	442.12	A	338.60	B	243.63	C	109.85	D	***

DW: dry weight. Mean values with different letters in the same column present significant differences. Significant level: ns = not significant. * = *p* < 0.05. ** = *p* < 0.01. *** = *p* < 0.001. <LOD: less than limit of detection.

## Data Availability

The data presented are included within the article and in the Supplementary Materials.

## Supplementary Materials

The following supporting information can be downloaded at: https://www.mdpi.com/article/10.3390/plants12152780/s1, Table S1: UHPLC-HRMS characteristics of phenolic compounds in banana samples.
