# Supplementary material for: Effects of Different Heating Treatments on the Antioxidant Activity and Phenolic Compounds of Ecuadorian Red Dacca Banana
Source: Plants (Basel). 2023 Jul 27;12(15):2780. doi: 10.3390/plants12152780 (PMC10420799; doi:10.3390/plants12152780)
Supplement: Supplementary file 1 [file plants-12-02780-s001.zip › plants-2509897-supplementary.pdf]

Table S1. UHPLC-HRMS characteristics of phenolic compounds in banana samples.

RT	Compounds	Chemical Formula	[m/z]- theoretical	Δ (ppm)	MSIMI level
	Flavan-3-ols				
9	**(+)-Catechin**	C15H14O6	289.0706	-1.123	1
9.6	**(-)-Epicatechin**	C15H14O6	289.0706	-0.258	1
8.3	**(-)-Epigallocatechin**	C15H14O7	305.0655	-0.194	1
11.8	**(+)-Gallocatechin**	C15H14O7	305.0655	-0.489	2
9.2	**Procyanidin B1**	C30H26O12	577.1323	-1.027	1
8.7	**Procyanidin B2**	C30H26O12	577.1323	-0.888	1
11	**Procyanidin dimer**	C30H26O12	577.1323	-1.095	2
8.9	**Procyanidin trimer I**	C45H38O18	865.1974	-1.468	2
9.8	**Procyanidin trimer II**	C45H38O18	865.1974	-1.538	2
	Flavanones				
12.8	**Eriodyctiol**	C15H12O6	287.0550	0.333	2
9.4	**Eriodyctiol hexoside I**	C21H22O11	449.1078	-0.864	2
9.7	**Eriodyctiol hexoside II**	C21H22O11	449.1078	-0.730	2
10.6	**Eriodyctiol hexoside III**	C21H22O11	449.1078	-0.440	2
10.4	**Eriodyctiol rutinoside**	C27H32O15	595.1657	-0.263	2
12.1	**Naringenin-7-glucoside**	C21H22O10	433.1129	-1.070	1
10.7	**Naringenin hexoside I**	C21H22O10	433.1129	-0.585	2
10.9	**Naringenin hexoside II**	C21H22O10	433.1129	-0.792	2
12.9	**Naringenin hexoside III**	C21H22O10	433.1129	-1.208	2
	Flavonols				
9.1	**Quercetin hexoside I**	C21H20O12	463.0877	-1.517	2
11.4	**Quercetin hexoside II**	C21H20O12	463.0877	-0.415	2
10.7	**Rutin**	C27H30O16	609.1440	0.819	1
10.1	**Quercetin dihexoside**	C27H30O17	625.1399	-0.057	2
10	**Quercetin 3-rutinoside 7-rhamnoside**	C33H40O20	755.2029	-1.509	2
8.3	**Quercetin trihexoside I**	C33H40O22	787.1938	-2.095	2
9.8	**Quercetin trihexoside II**	C33H40O22	787.1938	-1.320	2
11.8	**Isorhamnetin hexoside**	C22H22O12	477.1039	-1.158	2
11.5	**Isorhamnetin 3-rutinoside**	C28H32O16	623.1616	-2.088	2
11.2	**Kaempferol 3-rutinoside**	C27H30O15	593.1514	-0.331	1
10.4	**Kaempferol 3-rutinoside 7-rhamnoside**	C33H40O19	739.2056	-0.494	2
10.1	**Myricetin rutinoside**	C27H30O17	625.1381	-0.345	2
10.9	**Laricitrin 3-rutinoside**	C28H32O17	639.1536	-0.760	2
	Hydroxybenzoic acids				
2.5	**3,4,5-Trihydroxybenzoic acid**	C7H6O5	169.0131	-3.075	1
7.4	**3,4-dihydroxy-5-methoxybenzoic acid**	C8H8O5	183.0287	-2.294	1
8.7	**3,5-dihydroxy-4-methoxybenzoic acid**	C8H8O5	183.0287	-0.894	1
3	**3,4,5-Trihydroxybenzoic acid hexoside**	C13H16O10	331.0665	1.320	2
9.82	**3-Galloyl-Gallic acid**	C14H12O9	323.0390	0.222	2
11.4	**Syringaldehyde**	C9H10O4	181.0495	-2.791	2

8.2	**4-Hydroxybenzoic acid**	C7H6O3	137.0233	-3.945	1
9.6	**3-Hydroxybenzoic acid**	C7H6O3	137.0233	-4.529	1
4.5	**Hydroxybenzoic acid hexoside I**	C13H16O8	299.0767	-1.016	2
7	**Hydroxybenzoic acid hexoside II**	C13H16O8	299.0767	-0.314	2
6.6	**3,4-dihydroxybenzoic acid**	C7H6O4	153.0182	-3.889	1
8.3	**Dihydroxybenzoic acid**	C7H6O4	153.0182	-4.020	2
9	**4-Hydroxy-3-methoxybenzoic acid**	C8H8O4	167.0338	-3.084	1
10	**3-Hydroxy-4-methoxybenzoic acid**	C8H8O4	167.0338	-3.204	1
7.7	**3,5-dimethoxy-4-hydroxybenzoic acid hexoside I**	C15H20O10	359.0972	-0.287	2
8.3	**3,5-dimethoxy-4-hydroxybenzoic acid hexoside II**	C15H20O10	359.0972	0.103	2
	Hydroxycinnamic acids				
10.7	**Cinnamic acid**	C9H8O2	147.0440	-3.781	1
9.3	**3′,4′-dihydroxycinnamic acid**	C9H8O4	179.0338	-2.654	1
8	**Caffeoyl hexoside I**	C15H18O9	341.0867	-1.374	2
8.6	**Caffeoyl hexoside II**	C15H18O9	341.0867	-0.934	2
7.7	**Caffeoyl dihexoside**	C21H27O14	503.1395	-0.481	2
11.5	**4′-hydroxy-3′-methoxycinnamic acid**	C10H10O4	193.0495	-1.271	1
11.8	**3′-hydroxy-4′-methoxycinnamic acid**	C10H10O4	193.0495	-1.840	1
9.4	**4′-hydroxy-3′-methoxycinnamic acid hexoside**	C16H20O9	355.1023	0.595	2
8.7	**4′-hydroxy-3′-methoxycinnamic acid dihexoside I**	C22H30O14	517.1567	0.758	2
9.5	**4′-hydroxy-3′-methoxycinnamic acid dihexoside II**	C22H30O14	517.1567	-1.125	2
11.5	**4′-hydroxy-3′-methoxycinnamic acid conjugate**	C17H16O10	379.0666	-1.987	2
9.7	**3-(4′-hydroxyphenyl)propanoic acid**	C9H10O3	165.0546	-3.397	1
8.7	**3-(3′,4′-dihydroxyphenyl)propanoic acid**	C9H10O4	181.0495	-2.736	1
13	**Syringin**	C17H24O9	371.1326	-0.966	2
9.5	**Sinapic acid hexoside I**	C17H22O10	385.1129	-1.514	2
10.7	**Sinapic acid hexoside II**	C17H22O10	385.1129	-0.346	2
	Phenylacetic acids				
8.6	**Phenylacetic acid**	C8H8O2	135.0440	-3.969	1
9.5	**3-Hydroxyphenylacetic acid**	C8H8O3	151.0389	-3.513	1
7.7	**3,4-Dihydroxyphenylacetic acid**	C8H8O4	167.0338	-3.803	1
10.5	**4′-hydroxy-3′-methoxyphenylacetic acid**	C9H10O4	167.0338	-3.144	1
11	**3′-hydroxy-4′-methoxyphenylacetic acid**	C9H10O4	181.0495	-3.444	1

RT: retention time; Δ: mass error.
